# MELD-XI Score Is Associated With Short-Term Adverse Events in Patients With Heart Failure With Preserved Ejection

**DOI:** 10.3389/fcvm.2021.650191

**Published:** 2021-05-25

**Authors:** Sunying Wang, Yuwei Wang, Manqing Luo, Kaiyang Lin, Xiaoxu Xie, Na Lin, Qingyong Yang, Tian Zou, Xinan Chen, Xianwei Xie, Yansong Guo

**Affiliations:** ^1^Department of Cardiology, Shengli Clinical Medical College of Fujian Medical University, Fujian Provincial Hospital, Fuzhou, China; ^2^Fujian Yirong Information Technology Corporation, Fuzhou, China; ^3^Department of Epidemiology and Health Statistics, School of Public Health, Fujian Medical University, Fuzhou, China; ^4^Fujian Provincial Key Laboratory of Cardiovascular Disease, Fujian Provincial Center for Geriatrics, Fujian Clinical Medical Research Center for Cardiovascular Diseases, Fuzhou, China; ^5^Fujian Heart Failure Center Alliance, Fuzhou, China

**Keywords:** heart failure with preserved ejection fraction, MELD-XI score, prognosis, short-term, risk stratification

## Abstract

**Aim:** Accumulating evidence suggests that MELD-XI score holds the ability to predict the prognosis of congestive heart failure. However, most of the evidence is based on the end-stage heart failure population; thus, we aim to explore the association between the MELD-XI score and the prognosis in heart failure with preserved ejection fraction (HFpEF).

**Methods:** A total of 30,096 patients hospitalized for HFpEF in Fujian Provincial Hospital between January 1, 2014 and July 17, 2020 with available measures of creatinine and liver function were enrolled. The primary endpoint was 60-day in-hospital all-cause mortality. Secondary endpoints were 60-day in-hospital cardiovascular mortality and 30-day rehospitalization for heart failure.

**Results:** A total of 222 patients died within 60 days after admission, among which 75 deaths were considered cardiogenic. And 73 patients were readmitted for heart failure within 30 days after discharge. Generally, patients with an elevated MELD-XI score tended to have more comorbidities, higher NYHA class, and higher inflammatory biomarkers levels. Meanwhile, the MELD-XI score was positively correlated with NT-pro BNP, left atrial diameter, E/e' and negatively correlated with LVEF. After adjusting for conventional risk factors, the MELD-XI score was independently associated with 60-day in-hospital all-cause mortality [hazard ratio(HR) = 1.052, 95% confidential interval (CI) 1.022–1.083, *P* = 0.001], 60-day in-hospital cardiovascular mortality (HR = 1.064, 95% CI 1.013–1.118, *P* = 0.014), and 30-day readmission for heart failure (HR = 1.061, 95% CI 1.015–1.108, *P* = 0.009). Furthermore, the MELD-XI score added an incremental discriminatory capacity to risk stratification models developed based on this cohort.

**Conclusion:** The MELD-XI score was associated with short-term adverse events and provided additional discriminatory capacity to risk stratification models in patients hospitalized for HFpEF.

## Introduction

Patients with heart failure with preserved ejection fraction(HFpEF) account for 22–73% of the total population of heart failure (1). Compared with patients with heart failure with reduced ejection fraction(HFrEF), the prognosis of patients with HFpEF appears to be better, but there have not been many improvements throughout the years (2). Previous studies underscored that HFpEF is often accompanied by liver dysfunction and renal dysfunction, which are independent predictors for a poor prognosis (3–7). The mutual pathophysiological mechanism shared by HFpEF, impaired liver, and renal function is speculated to be the combination of insufficient perfusion, hormonal imbalance, and inflammation (3, 6, 8).

As an indicator of liver dysfunction and renal dysfunction, model of end-stage liver dysfunction (MELD-XI), has been shown to be negatively correlated with the survival rate in end-stage heart failure patients undergoing cardiac transplantation. It is used as a simple tool to assess appropriate candidates (9, 10). Furthermore, recent studies also suggested that the MELD-XI score is associated with poor prognosis in congestive and acute heart failure (11, 12).

To our knowledge, there is sparse evidence addressing the predictive value of the MELD-XI score in HfpEF, and this study may represent the largest real-world cohort to evaluate the utility of the MELD-XI score calculated at admission as a predictor for short-term prognoses in patients hospitalized for HFpEF.

## Methods

### Data Source and Study Definition

#### Study Population

A total of 30,096 patients hospitalized for HFpEF in Fujian Provincial Hospital between January 1, 2014 and July 17, 2020 with available measures of liver function were retrospectively enrolled. HFpEF was defined based on the 2016 ESC Guidelines for the diagnosis and treatment of acute and chronic heart failure, which is characterized by a left ventricular ejection fraction (LVEF) ≥50% (1). Patients with acute coronary syndrome, acute phase of stroke, advanced cancer, pregnancy, active rheumatic disease, advanced liver cirrhosis, and undergoing dialysis were excluded.

#### Endpoints

The primary endpoint was 60-day in-hospital all-cause mortality. Secondary endpoints were 60-day in-hospital cardiovascular mortality and 30-day rehospitalization for heart failure. The 30-day readmission for heart failure was defined as hospital admission for decompensated heart failure from 24 h to 30 days after discharge. In-hospital cardiovascular mortality was death, consistent with a ventricular tachyarrhythmia, and occurred in the absence of a known non-cardiac condition as the proximate cause of the death (13).

#### Biochemical Measurements

Liver function tests and renal function tests were quantified in a core laboratory (Roche, Modular-P chemical analyzer). Based on routine laboratory standards in Fujian Provincial Hospital, the upper limit was 23 μmol/L (1.35 mg/dl) for total bilirubin, similar to the standard published by previously studies (7).

The MELD-XI score was calculated as follows: 5.11 × Ln (total bilirubin as mg/dl)+11.76 × Ln (creatinine as mg/dl) + 9.44 (14). A MELD-XI score > 9.44 was considered elevated (12). eGFR was calculated using the simplified modification of diet in renal disease (MDRD) formula described by the National Kidney Foundation as follows: eGFR (ml/min/1.73 m^2^) = 175×(Scr)^−1.154^ × (Age)^−0.203^ × (0.742 if female) (15).

### Statistical Analysis

Continuous variables were presented as mean ± SD when normally distributed, as median and interquartile range (IQR) when skewed. Categorical variables were presented as frequencies and percentages. Differences between groups were evaluated by Student t, Mann-Whitney *U* test, or chi-square test as appropriate. The Spearman correlation test was applied to explore the association between the MELD-XI score and NT-pro BNP. A total of nine cox regression models were developed to estimate the association between the baseline MELD-XI score and the three study endpoints. Covariates in model 1 included age and gender. Covariates in model 2 included age, gender, elevated blood pressure (BP) at admission (>140/90mmHg), history of diabetes, history of atrial fibrillation, history of stroke, history of myocardial infarction, New York Heart Association (NYHA) class, LVEF, white blood cell count (WBC), triglyceride. Covariates in model 3 included age, gender, elevated BP at admission, history of diabetes, history of atrial fibrillation, history of stroke, history of myocardial infarction, NYHA class, NT-pro BNP, LVEF, WBC, triglyceride, use of digoxin, use of intravenous inotropes, use of beta-blocker, use of lipid-lowering agents. Models 1, 2, and 3 were used to estimate the risk of the three endpoints separately. Logistic regression and area under the receiver operating characteristic (ROC) curve were applied to evaluate the discriminatory capacity of the models for predicting 60-day all-cause in-hospital mortality. After adjusting for covariates stated above, age, LVEF, NYHA class, WBC, triglyceride remained independent risk factors, and thus were included in model 4.

These analyses were conducted using a statistical software package (SPSS version 25.0, IBM, Armonk, NY, USA). A *P*-value < 0.05 was considered statistically significant.

## Results

### Study Population

Participants had a mean age of 70.7 ± 12.8, 64.1% were male, 19,048(57.6%) had an ischemic etiology of heart failure, 1,627(5.4%) had history of liver disease, 2,299(7.6%) had elevated total bilirubin(>1.35 mg/dl), 673(2.2%) had a previous diagnosis of chronic kidney disease(CKD), and 9,715(32.3%)had an eGFR < 60 ml/min/1.73 m^2^.

Generally, patients with elevated MELD-XI score tended to be male, had worse cardiac function, and higher inflammatory biomarker levels. Furthermore, patients with elevated MELD-XI score were more likely to have previous diagnosis of hypertension and diabetes mellitus. However, patients with an elevated MELD-XI score seemed to receive less prescription of angiotensin-converting enzyme inhibitors (ACEI), angiotensin receptor blocker (ARB), angiotensin II type 1 receptor blockade and neprilysin inhibitor (ARNI), and mineralcorticoid receptor antagonist (MRA) (Table 1).

**Table 1:** Baseline characteristics between patients with elevated and normal MELD-XI score.

	**Elevated MELD-XI score**	**Normal MELD-XI score**	***P*-value**
	**(*n* = 3,429)**	**(*n* = 26,652)**	
**Demographics**			
Age(years)	67.5 ± 14.0	70.9 ± 12.6	<0.001
Gender(Male)%	2,330(67.9%)	16,741(62.8%)	<0.001
BMI(kg/m^2^)	23.5(21.0–26.0)	24.2(21.9–26.6)	<0.001
**Medical history**			
Hypertension(%)	3189(92.9%)	20803(78.1%)	<0.001
Diabetes(%)	1780(51.9%)	10325(38.7%)	<0.001
Atrial fibrillation(%)	345(10.1%)	3948(14.8%)	<0.001
Ischemic heart disease(%)	1212(35.3%)	16398(61.5%)	<0.001
Valvular heart disease(%)	399(11.6%)	3,477(13.0%)	0.02
Myocardial infarction(%)	296(8.6%)	2,507(9.4%)	<0.001
Stroke(%)	174(5.1%)	1,132(4.2%)	0.025
Chronic kidney disease(%)	464(13.5%)	209(0.8%)	<0.001
Liver disease(%)	297(8.7%)	1,329(5.0%)	<0.001
NYHA class 2(%)	1,138(38.0%)	17,520(70.7%)	<0.001
NYHA class 3(%)	1,156(38.6%)	5,707(23.0%)	
NYHA class 4(%)	704(23.5%)	1,562(6.3%)	
**Laboratory Measures**			
Troponin I(ng/ml)	0.060(0.027–0.142)	0.013(0.006–0.034)	<0.001
NT-pro BNP(pg/ml)	9,167(2,861–28,464)	369(101–1,446)	<0.001
HDL(mmol/L)	0.94 ± 0.40	1.10 ± 0.37	<0.001
LDL(mmol/L)	2.46 ± 1.13	2.57 ± 1.02	<0.001
Triglyceride(mmol/L)	1.73 ± 1.47	1.51 ± 1.15	<0.001
Total cholesterol(mmol/L)	4.13 ± 1.54	4.08 ± 1.19	0.049
WBC (10^9^/L)	6.48 ± 4.21	4.93 ± 2.89	<0.001
Hemoglobin(g/L)	89.72 ± 24.85	127.49 ± 22.41	<0.001
Platelet(10^9^/L)	200.64 ± 90.12	212.22 ± 80.18	<0.001
Na+(mmol/L)	137.11 ± 5.52	139.53 ± 4.64	<0.001
K+(mmol/L)	4.35 ± 0.82	4.05 ± 0.49	<0.001
CRP(mg/L)	23.1(5.3–78.1)	12.2(2.81–43.8)	<0.001
HbA1c (%)	6.37 ± 1.38	6.64 ± 1.39	<0.001
D-dimer(mg/L)	1.58(0.81–3.11)	0.58(0.30–1.28)	<0.001
**Echocardiography**			
LVEF	0.58 ± 0.04	0.59 ± 0.05	<0.001
Left Atrial Diameter(cm)	4.12(3.60–4.57)	3.72(3.36–4.21)	<0.001
E/e'	13.00 (10.00–17.4)	11.00 (8.83–14.00)	<0.001
**Medication**			
ACEIs/ARBs/ARNIs(%)	1,410(41.2%)	16,760(63.1%)	<0.001
MRAs(%)	574(16.8%)	6,732(25.3%)	<0.001
Diuretics(%)	2,536(74.0%)	10,279(38.7%)	<0.001
Digoxin(%)	448(13.1%)	3,590(13.5%)	0.481
I.v. inotropes(%)	568(16.6%)	4,120(15.5%)	0.104
Beta-blockers(%)	1,677(48.0%)	12,725(47.9%)	0.246
Lipid-Lowering Agents(%)	2,283(66.6%)	17,396(65.5%)	0.179
Nitrates(%)	1,128(32.9%)	8,430(31.7%)	0.158
Anticoagulants(%)	1,851(54%)	14,088(53%)	0.27

*BMI, body mass index; LVEF, left ventricular ejection fraction; NYHA class, New York Heart Association class; NT-pro BNP, N-terminal pro brain natriuretic peptide; HDL, high density lipoprotein; LDL, low density lipoprotein; WBC, white blood cell count; CRP, C-reactive protein; ACEI, angiotensin-converting enzyme inhibitors; ARB, angiotensin receptor blocker; ARNI, angiotensin II type 1 receptor blockade and neprilysin inhibitor; MRA, mineralcorticoid receptor antagonist*.

### Association Between Cardiac Function and MELD-XI Score

Patients with elevated MELD-XI scores had significantly higher NT-pro BNP levels (Median = 9,167 pg/ml vs. 369 pg/ml, *P* < 0.001), higher left atrial diameter (Median = 4.12 cm vs. 3.72 cm, *P* < 0.001), higher E/e' (Median = 13.00 vs. 11.00, *P* < 0.001) and lower LVEF (0.58 ± 0.04 vs. 0.59 ± 0.05, *P* < 0.001) (Table 1). The MELD-XI score had a moderate correlation with NT-pro BNP (Spearman rho = 0.497, *P* < 0.001) and a weak correlation with left atrial diameter (Spearman rho = 0.209, *P* < 0.001) and E/e' (Spearman rho = 0.090, *P* < 0.001). A weak and inverse correlation was also determined between the MELD-XI score and LVEF (Spearman rho = −0.084, *P* < 0.001) (Figure 3).

### MELD-XI Score and Short-Term Adverse Events

**Figure 1:**
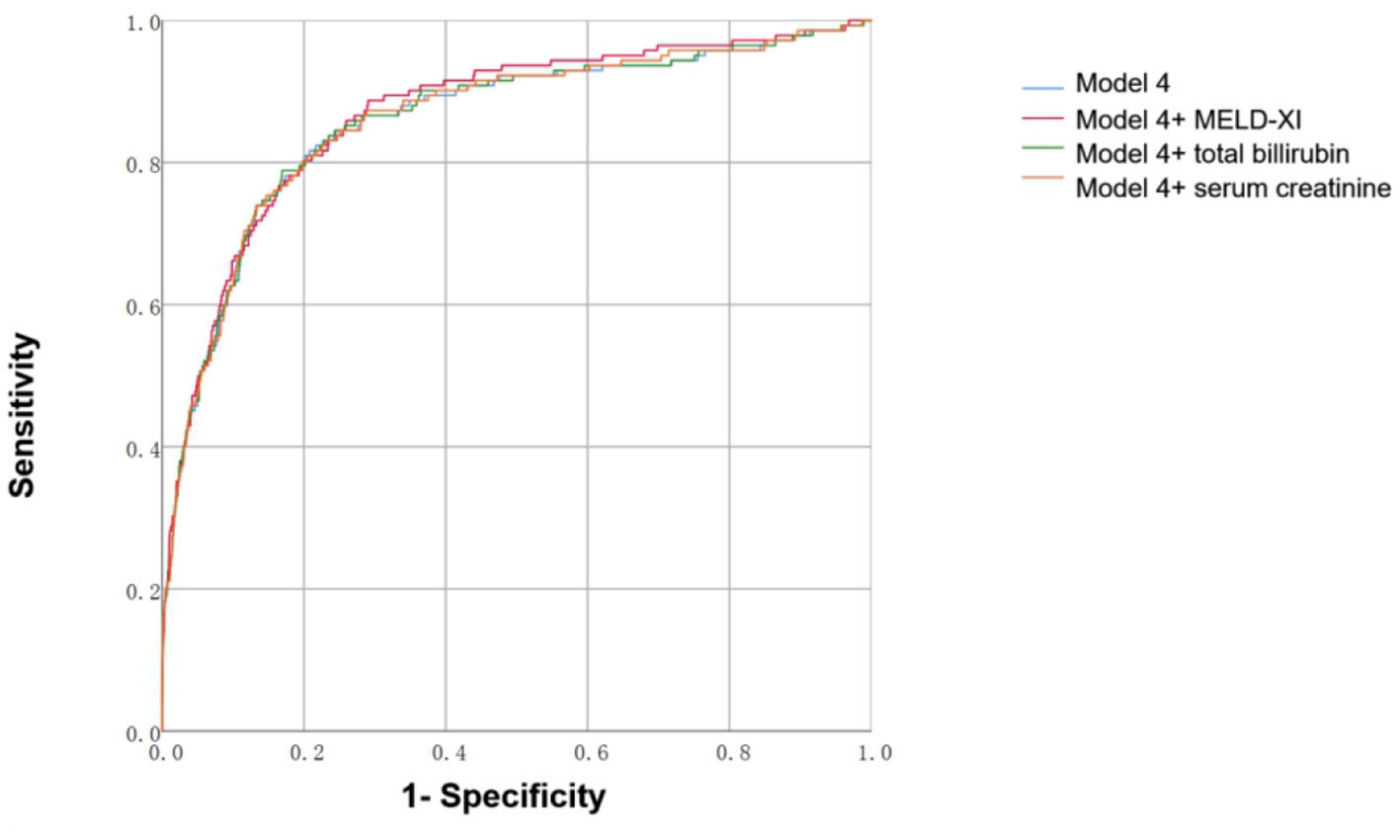
Kaplan–Meier estimates of short-term clinical outcomes of according to baseline MELD-XI score.

A total of 222 patients died within 60 days after admission, among which 75 deaths were considered cardiogenic. And among 29,844 living patients within 30 days after discharge, 73 were readmitted for heart failure within 30 days after discharge. Patients with elevated MELD-XI scores suffered a significantly higher risk of mortality, cardiovascular mortality, and readmission for heart failure compared with those with normal MELD-XI (Figure 1). After adjusting for conventional risk factors, the MELD-XI score remained a predominant predictor for 60-day in-hospital all-cause mortality (HR = 1.052, 95%CI 1.022–1.083, *P* = 0.001), 60-day in-hospital cardiovascular mortality (HR = 1.064, 95% CI 1.013–1.118, *P* = 0.014), and 30-day readmission **(**HR = 1.061, 95% CI 11.015–1.108, *P* = 0.009) (Table 2).

**Table 2:** Risk of short-term events for each 1-point increase in MELD-XI score.

	**HR(95% CI)**	***P*-value**
**60-Day In-Hospital All-Cause Mortality**		
Model 1	1.126 (1.090–1.164)	<0.001
Model 2	1.115 (1.093–1.093)	0.001
Model 3	1.052 (1.022–1.083)	0.001
**60-Day In-Hospital Cardiovascular Mortality**		
Model 1	1.125 (1.087–1.163)	<0.001
Model 2	1.063 (1.012–1.117)	0.015
Model 3	1.064 (1.013–1.118)	0.014
**30-Day Readmission**		
Model 1	1.108 (1.069–1.148)	<0.001
Model 2	1.064 (1.018–1.111)	0.006
Model 3	1.061 (1.015–1.108)	0.009

***Model 1:** Adjusted for age and gender*.

***Model 2:** Adjusted for age, gender, BP at admission >140/90 mmHg, history of diabetes, history of atrial fibrillation, history of stroke, history of myocardial infarction, New York Heart Association (NYHA) class, left ventricular ejection fraction (LVEF), white blood cell count, triglyceride*.

***Model 3:** Adjusted for age, gender, BP at admission >140/90 mmHg, history of diabetes, history of atrial fibrillation, history of stroke, history of myocardial infarction, NYHA class, LVEF, white blood cell count, triglyceride, use of digoxin, use of intravenous inotropes, use of beta-blocker, use of lipid-lowering agents*.

### Predictive Value of Models Includes MELD-XI

**Figure 2:**
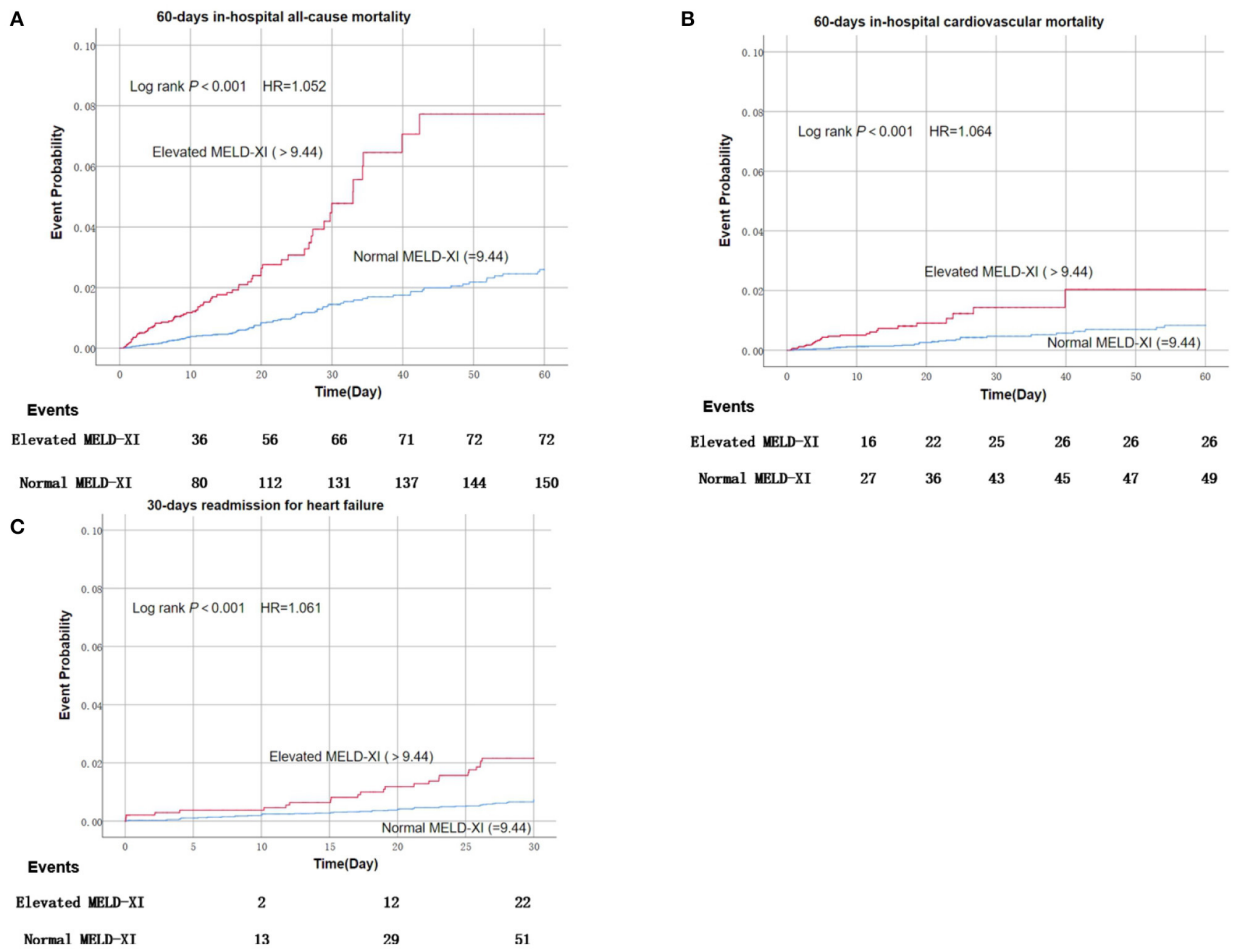
**(A-C)** Area under the receiver operating characteristic curve of models predicting 60-day in-hospital all-cause mortality and cumulative events. Model 4: Adjusted for age, LVEF, NYHA class, WBC, triglyceride. Calculated as: −0.015 + 0.019 × age −14.604 × LVEF + 1.293 × NYHA class III (1) [OR 2.871 × NYHA Class IV (1)] + 0.046 × WBC(10^9^/L) + 0.118 × triglyceride(mmol/L). Model 4+MELD-XI: −0.420 + 0.024 × age −14.488 × LVEF + 1.135 × NYHA class III (1) [OR 2.567 × NYHA Class IV (1)] + 0.043 × WBC(10^9^/L) + 0.109 × triglyceride(mmol/L) + 0.053 × MELD-XI. Model 4+serum creatinine: −0.163 + 0.021 × age −14.593 × LVEF + 1.267 × NYHA class III (1) [OR 2.830 × NYHA Class IV (1)] + 0.046 × WBC(10^9^/L) + 0.116 × triglyceride(mmol/L) + 0.031 × serum creatinine(mg/dl). Model 4+total bilirubin: −0.128 + 0.020 × age −14.639 × LVEF + 1.282 × NYHA class III (1) [OR 2.842 × NYHA Class IV (1)] + 0.044 × WBC(10^9^/L) + 0.118 × triglyceride(mmol/L) + 0.083 × total bilirubin (mg/dl).

We constructed risk stratification models for prediction of in-hospital all-cause mortality. As shown in Figure 2 and Table 3, adding the MELD-XI score slightly increased the discriminatory capacity of model 4 (AUC = 0.868 vs. 0.858, *P* = 0.0162), which was better than models including total bilirubin or serum creatinine (AUC = 0.868 vs. 0.859, *P* = 0.0296; AUC = 0.868 vs. 0.860, *P* = 0.0161).

**Figure 3:**
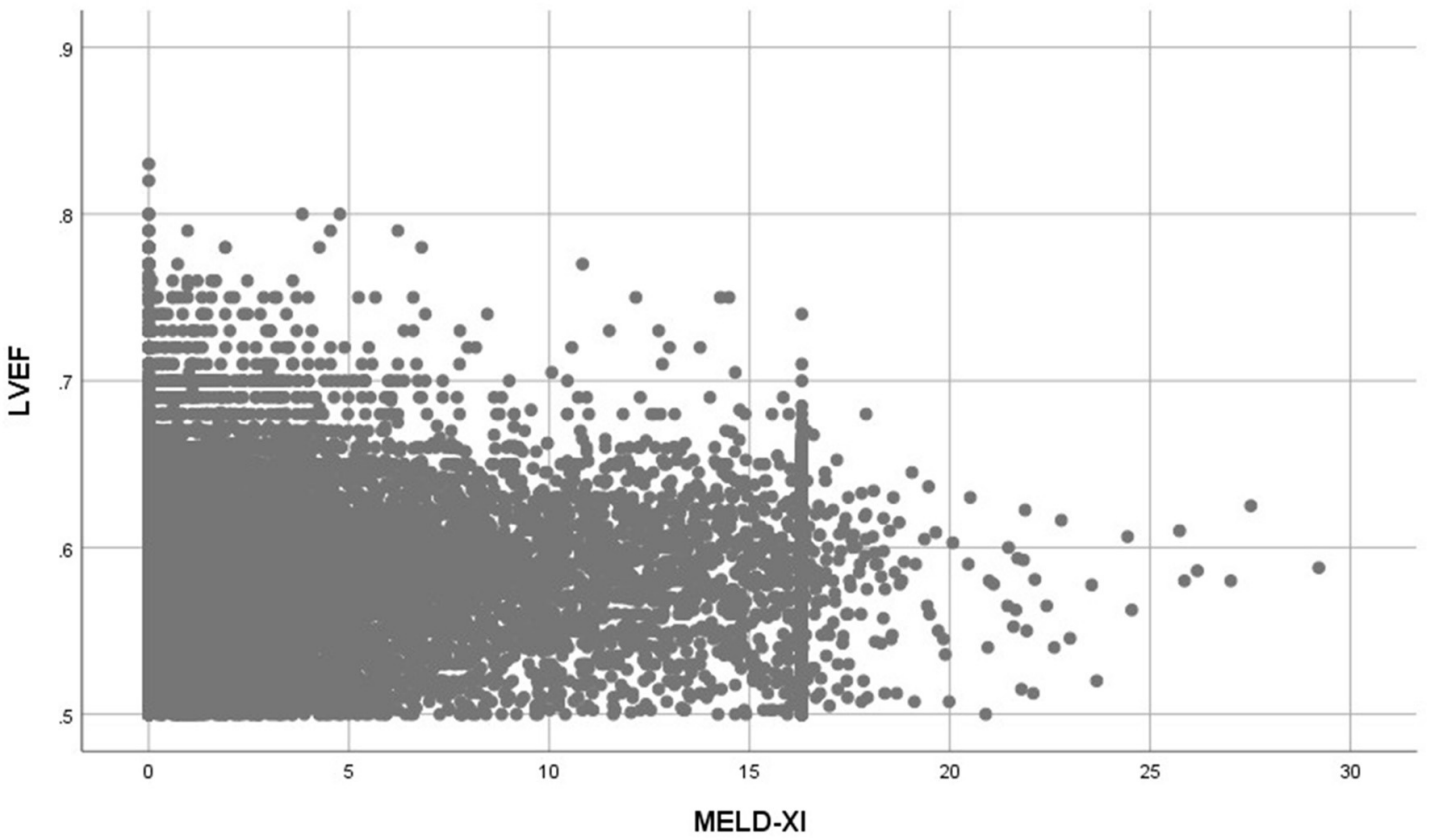
Scatter plot between LVEF and MELD-XI score.

**Table 3:** AUC of models predicting 60-day in-hospital all-cause mortality.

	**AUC (95% CI)**	***P*-value**
Model 4	0.858 (0.821–0.894)	<0.001
Model 4+ MELD-XI	0.868 (0.835–0.901)	<0.001
Model 4+ total bilirubin	0.859 (0.823–0.895)	<0.001
Model 4+ serum creatinine	0.86 (0.824–0.895)	<0.001

## Discussion

It has been noticed that both liver dysfunction and renal dysfunction are common comorbidities during congestive heart failure as well as associated with ominous prognosis (3–7). As a combined index of liver function and renal function, it has been shown that the MELD-XI score holds the ability to predict adverse events in patients with acute heart failure and end-stage heart failure (11, 12). To our knowledge, this study is the first to find the MELD-XI score to be a risk factor for short-term adverse events, improving risk stratification models for patients hospitalized with HFpEF.

The predictive ability of liver dysfunction and renal dysfunction have been confirmed in heart failure. A study by Allen et al. (16) demonstrated that abnormal liver function tests are prevalent in patients with heart failure, and total bilirubin could be one of the most powerful predictors for poor prognosis among all liver function tests. Furthermore, Prenner et al. (5) conducted a *post-hoc* study of TOP-CAT trial and indicated that albumin was also a strong predictor of adverse events in patients with HFpEF. Data from a most recent *post-hoc* study of TOP-CAT suggested that not only kidney function tests but also their variability between visits are independently correlated with clinical outcomes (17).

As a modification of the MELD score, the MELD-XI score excludes international normalized ratio and consists of only total bilirubin and serum creatinine, thus could be applied to heart failure patients receiving anticoagulants. Previous investigators confirmed that higher MELD-XI value is associated with mortality in patients with advanced heart failure undergoing heart transplantation, thus could serve as a simple quantitative tool for screening appropriate candidates receiving heart transplantation (11, 18). Afterward, the MELD-XI score was also validated in patients with acute heart failure as an innovative prognosticator (12, 19). Recently Abe et al. (20) retrospectively analyzed 562 patients with decompensated heart failure and concluded that MELD-XI was an independent predictor of mortality in general heart failure patients. In our cohort, 53% patients received anticoagulants; hence, MELD-XI seems an appropriate approach to estimate liver and renal function compared to original MELD score and MELD-Na score. And to extend this conclusion from previous study, we analyzed short-term events separately and confirmed the prognostic role of MELD-XI in the setting of HFpEF.

Analyzing each possible risk factor is the foundation of accurate risk stratification, which ultimately contributes to the management strategy of patients with HFpEF (1). A Seattle Heart Failure Model was derived from the population of general heart failure then validated in 9,942 patients, which provided an overall AUC of 0.729 (21). The MUSIK risk score, which contains 10 variables (eGFR), was developed in a cohort of 992 ambulatory heart failure patients and demonstrated good performance of predicting mortality (22). Risk stratification models developed in our study contained only six variables and yielded good discriminatory capacity for short-term adverse events, which was improved slightly by adding the MELD-XI score.

The pathophysiological connection between MELD-XI and HFpEF might consist of several aspects. First, decompensated cardiac function provides insufficient perfusion to both the kidneys and liver, thus leading to decreased glomerular filtration rate as well as tissue damage of renal and liver (6, 8, 23). Second, elevated right atrial pressure due to heart failure, especially HFpEF, usually results in passive hepatic venous congestion, lipid metabolism disorder, and even cirrhosis (4, 23–25). Third, neurohumoral disorder results in vascular contractility disorder in HfpEF, which further reduces renal blood flow and liver perfusion (25, 26). And in turn, impaired liver and renal function contribute to hemodynamic changes and inflammatory activity, eventually resulting in cardiac stiffness (3, 6, 24). Data from our study have confirmed the association between MELD-XI and NT-pro BNP, which reinforced that volume overload is the reflexion of the vicious cycle formed by HFpEF and impaired liver and renal function. And we also found that MELD-XI was correlated with cardiac function indexes derived from echocardiography, which supports that impaired liver and renal function is responsible for the deteriorated cardiac stiffness during HFpEF.

Our study demonstrated that the MELD-XI score not only was associated with short-term adverse events but also offered increased discriminatory ability for risk stratification models in patients hospitalized for HFpEF.

## Limitation

First, as a retrospective study based on the data of a single center, the conclusion should be further validated in different regions and different populations. Second, we failed to analyze the association between long-term prognosis and the MELD-XI score since we have not been able to complete the follow-up procedure due to the large scale of this study, although it is still crucial to focus on the risk stratification of long-term events. Third, considering the extendibility of the conclusion, we only excluded patients with advanced liver cirrhosis and those undergoing dialysis, not all patients with previously diagnosed liver disease and renal disease.

## Data Availability Statement

The raw data supporting the conclusions of this article will be made available by the authors, without undue reservation.

## Ethics Statement

The studies involving human participants were reviewed and approved by Fujian Provincial Hospital Ethics Committee (福绺省立猻癢缦理秔瑘缚). The patients/participants provided their written informed consent to participate in this study. Written informed consent was obtained from the individual(s) for the publication of any potentially identifiable images or data included in this article.

## Author Contributions

SW and YW built this large scale database containing the clinical information of heart failure patients in Fujian Provincial Hospital. SW and XiaoX conducted the statistical analysis. YG is the overseer of this study. Rest of the team contributed to the idea gathering. All authors contributed to the article and approved the submitted version.

## Conflict of Interest

YW is a software engineer employed by Fujian Yirong Information Technology Corporation and she provided her information technological support for free. The remaining authors declare that the research was conducted in the absence of any commercial or financial relationships that could be construed as a potential conflict of interest.
