# Corrigendum: MELD-XI Score Is Associated With Short-Term Adverse Events in Patients With Heart Failure With Preserved Ejection

**DOI:** 10.3389/fcvm.2021.717166

**Published:** 2021-06-25

**Authors:** Sunying Wang, Yuwei Wang, Manqing Luo, Kaiyang Lin, Xiaoxu Xie, Na Lin, Qingyong Yang, Tian Zou, Xinan Chen, Xianwei Xie, Yansong Guo

**Affiliations:** ^1^Department of Cardiology, Shengli Clinical Medical College of Fujian Medical University, Fujian Provincial Hospital, Fuzhou, China; ^2^Fujian Yirong Information Technology Corporation, Fuzhou, China; ^3^Department of Epidemiology and Health Statistics, School of Public Health, Fujian Medical University, Fuzhou, China; ^4^Fujian Provincial Key Laboratory of Cardiovascular Disease, Fujian Provincial Center for Geriatrics, Fujian Clinical Medical Research Center for Cardiovascular Diseases, Fuzhou, China; ^5^Fujian Heart Failure Center Alliance, Fuzhou, China

**Keywords:** heart failure with preserved ejection fraction, MELD-XI score, prognosis, short-term, risk stratification

There is an error in the title. The correct title for “MMMELD-XI Score Is Associated With Short-Term Adverse Events in Patients With Heart Failure With Preserved Ejection Fraction” is “MELD-XI Score Is Associated With Short-Term Adverse Events in Patients With Heart Failure With Preserved Ejection.”

In the published article, there was an error regarding the affiliation(s) for Xiaoxu Xie. Instead of “Department of Cardiology, Shengli Clinical Medical College, Fujian Medical University, Fuzhou, China,” it should be “Department of Epidemiology and Health Statistics, School of Public Health, Fujian Medical University, Fuzhou, China.” In the published article, there was also an error regarding the affiliation(s) for Yuwei Wang. Instead of “Department of Cardiology, Shengli Clinical Medical College, Fujian Medical University, Fuzhou, China,” it should be “Fujian Yirong Information Technology Corporation, Fuzhou, China.” In the published article, there was an error regarding the affiliation(s) for Yansong Guo, Sunying Wang, Manqing Luo, Kaiyang Lin, Na Lin, Qingyong Yang, Tian Zou, Xinan Chen, and Xianwei Xie. Instead of affiliation(s) “Department of Cardiology, Shengli Clinical Medical College, Fujian Medical University, Fuzhou, China,” it should be “Department of Cardiology, Shengli Clinical Medical College of Fujian Medical University, Fujian Provincial Hospital, Fuzhou, China.”

In the published article, there was an error regarding the affiliation(s) for Yansong Guo. As well as having affiliation 1 “Department of Cardiology, Shengli Clinical Medical College of Fujian Medical University, Fujian Provincial Hospital, Fuzhou, China,” they should also have affiliation 4 “Fujian Provincial Key Laboratory of Cardiovascular Disease, Fujian Provincial Center for Geriatrics, Fujian Clinical Medical Research Center for Cardiovascular Diseases, Fuzhou, China,” and 5 “Fujian Heart Failure Center Alliance, Fuzhou, China.”

The authors apologize for these errors and state that this does not change the scientific conclusions of the article in any way. The original article has been updated.

